# Comet Lesions in Patients with Pseudoxanthoma Elasticum

**DOI:** 10.4274/tjo.84756

**Published:** 2015-12-05

**Authors:** Sinan Tatlıpınar, Berna Şahan, Muhsin Altunsoy

**Affiliations:** 1 Yeditepe University Faculty of Medicine, Department of Ophthalmology, İstanbul, Turkey

**Keywords:** Pseudoxanthoma elasticum, comet lesions, angioid streaks

## Abstract

Pseudoxanthoma elasticum (PXE) is a genetic multisystemic disorder affecting the skin, eyes and cardiovascular system. Basic fundoscopic findings in PXE result from Bruch’s membrane involvement. The most important fundoscopic findings are angioid streaks. Other significant ocular findings are peau d’orange appearance, optic disc drusen, pattern dystrophy-like macular appearance, comet lesions, and choroidal neovascularization. Comet lesions are a pathognomonic ocular finding for PXE. The presence of both angioid streaks in the fundus and typical skin lesions should alert clinicians to PXE. Herein, we present two PXE cases with comet lesions.

## INTRODUCTION

Pseudoxanthoma elasticum (PXE) is a genetic multisystemic disorder affecting the skin, cardiovascular system and eyes, and is characterized by fragmentation and mineralization of elastic fibrils.^1^ The reported incidence of PXE varies between 1/25,000 and 1/100,000.^2^

The basic fundoscopic findings in PXE arise as a result of Bruch’s membrane involvement.^1^ The most important fundus finding is angioid streaks. Other important ocular findings include peau d’orange (orange peel) appearance, optic disc drusen, pattern dystrophy-like appearance, comet lesions and choroidal neovascularization (CNV).^3,4^

This case report presents the fundoscopic findings of two cases of PXE, including the lesser known, pathognomonic finding of comet lesions.

## CASE REPORTS

### Case 1

A 15-year-old male patient was referred to our clinic due to fundoscopic findings. Best corrected visual acuity was 1.0 in both eyes (Snellen). Slit-lamp examination of the anterior segment and intraocular pressures were within normal limits. Fundoscopic examination revealed angioid streaks around the optic discs and peau d’orange appearance in the temporal macula of both eyes. Optic disc drusen were present in both eyes, but were more distinct in the left eye.

**Figure 1:**
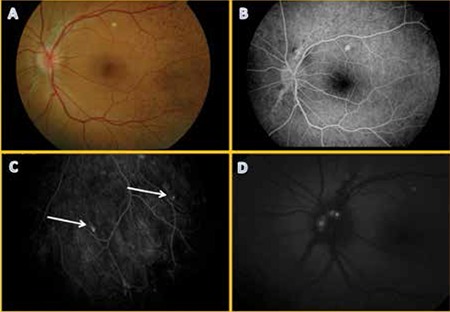
Case 1, fundus photography of 15-year-old male patient revealed (A) peau d’orange in the temporal macula; (B) peripapillary angioid streaks and (C) comet lesions (white arrows) were clearly visible on fundus fluorescein angiography; (D) autofluorescence photography showed typical optic disc drusen appearance

Fundus fluorescein angiography (FFA) revealed angioid streaks and comet lesions in both eyes. Optic disc drusen was visible in the left eye on fundus autofluorescence imaging (Figure 1). Suspecting PXE, dermatology and cardiology consultations were requested. Biopsy of skin lesions taken during dermatologic examination confirmed PXE.

### Case 2

**Figure 2:**
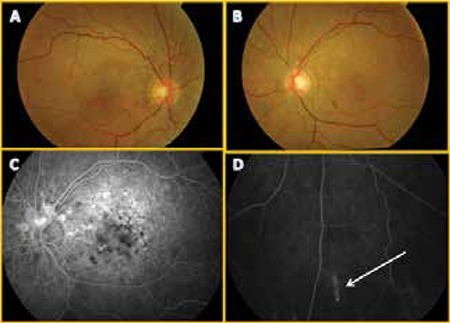
Case 2, fundus photography of 47-year-old female patient revealed (A, B) bilateral angioid streaks, subretinal drusenoid structures and (B) subretinal hemorrhage inferonasal to the fovea in the left eye; fundus fluorescein angiography revealed (C) peripapillary angioid streaks and (D) typical comet lesion in the inferior periphery (white arrow)

A 47-year-old female patient presented to our clinic with complaints of decreased vision in her left eye. Her best corrected visual acuity was 1.0 in the right eye and 0.8 partial in the left eye (Snellen). Slit-lamp examination of the anterior segment and intraocular pressures were within normal limits. Fundoscopic examination revealed angioid streaks in both eyes, and subretinal fluid in the foveal area and subretinal hemorrhage inferonasal to the fovea in the left eye. Optical coherence tomography (OCT) revealed subfoveal fluid in the left eye. FFA showed bilateral angioid streaks and comet lesions, as well as CNV in the left eye (Figure 2).

The patient was given two intravitreal anti-VEGF injections for the CNV secondary to angioid streaks. The patient continues to be followed in our clinic and has shown improvement in visual acuity.

## DISCUSSION

PXE shows an autosomal recessive inheritance pattern. There are over 200 reported mutations of the PXE gene ABCC6, located on chromosome 16.^5^

PXE is a multisystemic disorder involving the skin, cardiovascular system and eyes characterized by fragmentation and calcification of elastic fibrils. PXE leads to calcification of Bruch’s membrane and retinal pigment epithelium (RPE) atrophy in the fundus. The resulting angioid streaks, characterized by irregular red-brown and gray lines extending peripherally from the peripapillary area, are the most common funduscopic finding of PXE.^6^ PXE with angioid streaks is also known as Groenblad-Strandberg syndrome.^7^ Angioid streaks are not specific to PXE and may occur due to many systemic diseases, including sickle-cell anemia, beta thalassemia, Paget’s disease, and Marfan and Ehler-Danlos syndromes.^1,8,9^ CNV may occur in association with angioid streaks and may lead to serious vision loss.^10^ Intravitreal anti-VEGF therapy has been shown to improve visual acuity in PXE patients with CNV.^11^

The earliest fundus finding of PXE is peau d’orange, which occurs as the result of the accumulation of yellow material in the RPE and is seen more in the temporal macula.1 Angioid streaks and peau d’orange are among the major diagnostic criteria of PXE; comet lesions are one of the minor criteria.^1^ Comet lesions appear on FFA as approximately 125 microns in size in the mid-periphery and includes a solitary, subretinal, nodular, atrophic white spot followed by a wedge-shaped atrophic area pointing toward the optic disc.^1,8^ Its distinctive FFA appearance is very well-known and the lesion shows hyperfluorescence.^4^ This finding is pathognomonic for PXE.^1,12^ As comet lesions generally appear in the periphery, their effect on vision is unknown.^4^ In some cases, several comet lesions appear together, which is called a comet shower.^13^ In a study by De Zaeytijd et al.^13^ including 22 PXE patients, comet lesions were observed in 90% of the cases. Plomp et al.^14^ found comet lesions in 60% of 15 homozygous PXE patients.

Angioid streaks and comet lesions were present in both of our cases. One of the patients had peau d’orange and optic disc drusen, while the other had CNV.

For both of our patients, ophthalmologic examination was followed by a dermatology consultation and the patients’ skin lesions were biopsied. Pathology results confirmed PXE.

The primary organ affected by PXE is the skin. Skin lesions first appear around 13 years of age.^15,16^ The lesions appear on the neck, axilla and poplitea and are small and yellow-ivory in color with a reticular pattern. However, a diagnosis of PXE cannot be eliminated in the absence of skin lesions.^17^ In the cardiovascular system, PXE has been reported to lead to angina pectoris, arterial hypertension, restrictive cardiomyopathy, mitral valve prolapse and stenosis, heart failure and sudden cardiac death.^3^ Gastrointestinal hemorrhages tend to be recurrent and their severity may be life-threatening.^3^ Angioid streaks are not typically seen in PXE patients under 10 years old, but are present in nearly all patients 20 years after diagnosis.^18^

PXE should be considered for patients with the fundoscopic finding of angioid streaks. The presence of skin lesions supports this diagnosis. A dermatology consultation to evaluate patients’ skin lesions, and cardiology and internal medicine consultations to investigate cardiovascular and gastrointestinal involvement should be requested. If necessary, skin lesions should be biopsied to confirm the diagnosis.
